# Complete Genome Sequence of Clostridium cadaveris IFB3C5, Isolated from a Human Colonic Adenocarcinoma

**DOI:** 10.1128/mra.01135-21

**Published:** 2022-03-02

**Authors:** Adam S. McGlinchey, Martha A. Zepeda-Rivera, Marija Stepanovica, Alexander A. Baryiames, Dakota S. Jones, Kaitlyn D. LaCourse, Susan Bullman, Christopher D. Johnston

**Affiliations:** a Vaccine and Infectious Disease Division, Fred Hutchinson Cancer Research Center, Seattle, Washington, USA; b Human Biology Division, Fred Hutchinson Cancer Research Center, Seattle, Washington, USA; University of Rochester School of Medicine and Dentistry

## Abstract

We report the complete genome sequence of Clostridium cadaveris IFB3C5, a strain isolated from the resected tumor of a treatment naive colorectal cancer patient. This genome is comprised of a singular chromosome of approximately 3.63 Mbp in length, contains two plasmids, and has an overall mean GC content of 31.7%.

## ANNOUNCEMENT

Clostridium cadaveris, first isolated in 1899 (1), is a rod-shaped, Gram-positive anaerobic bacterium typically present in the human gastrointestinal tract (2, 3). Reported pathogenic associations include equine idiopathic colitis (4), a human abscess (5), bacteremia (6), and chronic osteomyelitis (7). Here, we report the isolation of C. cadaveris IFB3C5, a strain cultivated from the necrotic tissue of a colorectal cancer tumor.

**FIG 1:**
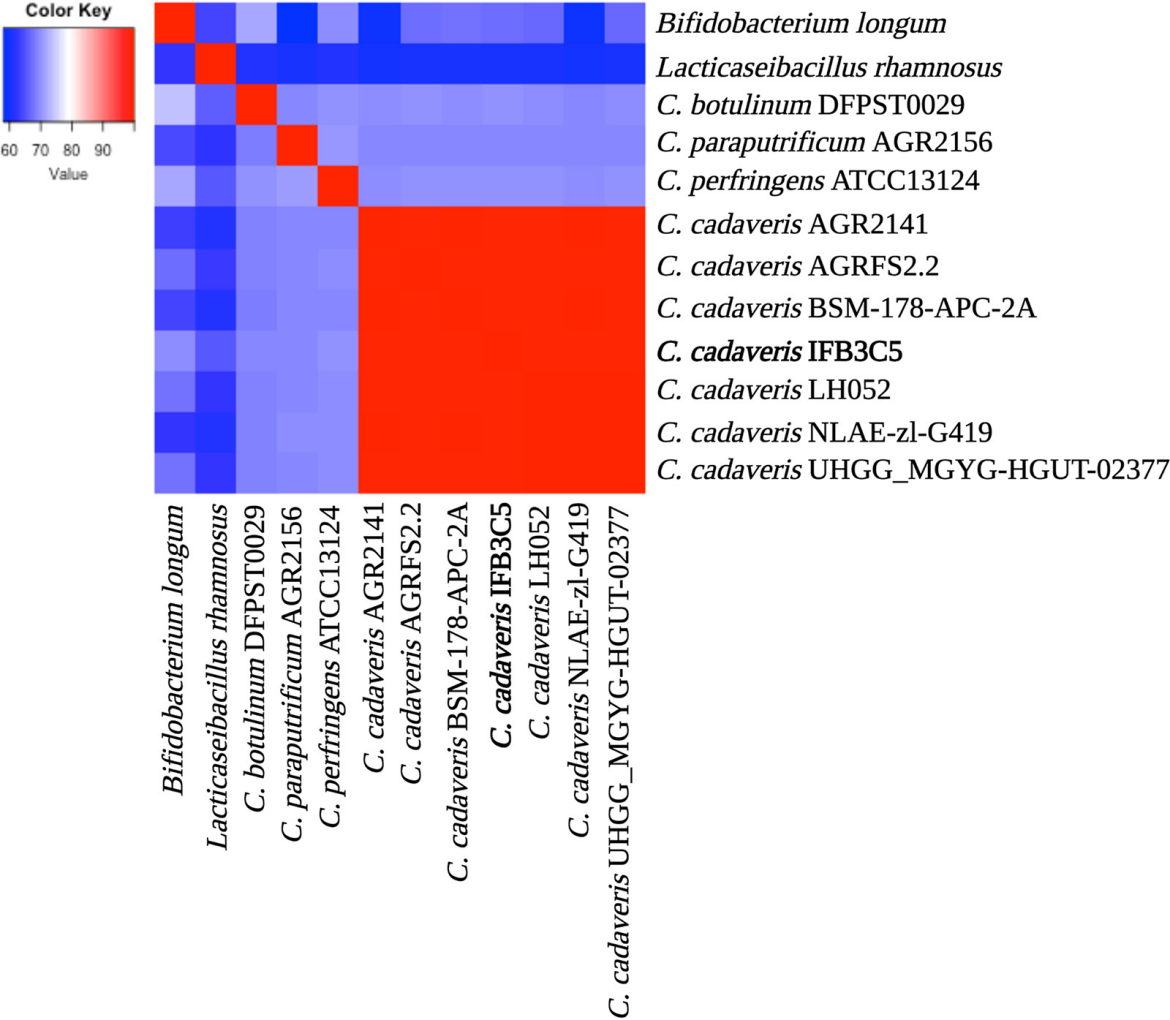
Heat map of average nucleotide identity (ANI) values. The genome of *C. cadaveris* IFB3C5 was compared to publicly available genomes of six additional *C. cadaveris* strains, three different *Clostridium* species, and two outgroups, i.e., Bifidobacterium longum and Lacticaseibacillus rhamnosus (16) (Table 1), using JSpeciesWS (17). Red indicates a higher ANI value, whereas blue indicates a lower ANI value. *C. cadaveris* IFB3C5 had an ANI score above 99% against each *C. cadaveris* strain, scores of 68 to 70% against other species of *Clostridium*, and scores of 59 to 68% against B. longum and L. rhamnosus outgroups (16). The heat map was generated using the heatmap.2 function from the gplots package on RStudio (version 1.4.1103) (18). The final figure was created on BioRender.

*C. cadaveris* IFB3C5 was isolated from a cryopreserved colon adenocarcinoma of a 67-year-old treatment-naive female colorectal cancer patient, originally resected in 1989 in Seattle, WA. Classification as *C. cadaveris* is based on 16S rRNA gene sequencing and average nucleotide identity analysis (Table 1 and Fig. 1). *C. cadaveris* IFB3C5 was cultured under anaerobic conditions (Oxoid, Thermo Fisher Scientific, USA). High-molecular-weight genomic DNA was extracted using the MasterPure DNA purification kit (Epicentre, Lucigen, USA). Single-molecule real-time sequencing (SMRT-Seq) (8) was carried out on a PacBio Sequel I instrument (Pacific Biosciences, USA). QuBit double-stranded DNA (dsDNA) broad-range (BR) assays (Thermo Fisher Scientific, USA), determined the DNA concentration, and 3 μg of DNA was sheared to an average size of 12 kb using G-tube (Covaris, USA). Libraries were generated using the SMRTbell Express template prep kit 2.0 (Pacific Biosciences), and pooled libraries were size selected via the BluePippin system (Sage Sciences, USA) at a 4-kb minimum threshold. The Pacific Biosciences SMRTAnalysis pipeline version 9.0.0.92188 first processed sequencing reads and then assembled them using Microbial Assembler, which includes an error correction step for chromosomal contiguity and rotation to place the first nucleotide at the chromosomal replication gene, *dnaA*. Genome assembly showed 21,182 polymerase reads that were further partitioned into 195,640 subreads with an *N*_50_ value of 5,553 nucleotides and a total number of subread bases of 787,902,561 with a mean coverage of 212×. Genome assembly resulted in three contigs: a chromosomal sequence of 3,619,347 bp and two putative plasmids of 4,819 bp and 1,618 bp.

**TABLE 1:** Publicly available genome assemblies used for ANI analysis

Species	Strain	Accession no.	Isolation source
*C. cadaveris*	AGR2141	GCF_000424205.1	Rumen microbiome
*C. cadaveris*	BSM-178-APC-2A	GCF_012844035.1	Pig fecal sample
*C. cadaveris*	AGRFS2.2	GCF_013390975.1	Dairy farm
*C. cadaveris*	NLAE-zl-G419	GCF_900113105.1	
*C. cadaveris*	LH052	GCF_900217165.1	Human preterm infant fecal sample
Clostridium paraputrificum	AGR2156	GCF_000424025.1	Rumen microbiome
Clostridium perfringens	ATCC 13124	GCF_000013285.1	
Clostridium botulinum	DFPST0029	GCF_003058345.1	Contaminated food specimen
Bifidobacterium longum	51A	GCF_004936435.1	Human fecal sample
*Lacticaseibacillus rhamnosus*	UMB0004	GCF_002848015.1	Catheter

Genome annotation using the NCBI Prokaryotic Genome Annotation Pipeline (PGAP) (9) identified 3,392 coding sequences, a GC content of 31.7%, and 112 RNAs. Methylome annotation via the Restriction Enzyme Database (REBASE) (10) identified two putative restriction-modification (RM) systems, a type I RM system with the modified bipartite motif ACBN_6_TCTG and a type II RM system with the modified motif CRAAAAR. For the latter, a similar motif, CAAAAA, influences sporulation in the related organism Clostridioides difficile (11). Detection of RM systems prompted investigation into CRISPR defense systems. CRISPRDetect (12) and CRISPRCasTyper (13) analyses identified a type I-B CRISPR-Cas system with a 58-spacer array.

PlasMapper (14) identified replication-associated genes in both putative plasmids. Putative plasmids showed no significant similarity to each other via BLASTN alignment, supporting the notion that *C. cadaveris* IFB3C5 carries two distinct plasmids. Antimicrobial resistance gene detection via the Comprehensive Antibiotic Resistance Database (CARD) (15) identified a chromosomal variant in the *gyrB* gene, which encodes fluoroquinolone resistance.

Currently, seven incomplete *C. cadaveris* genome assemblies are publicly available. This first complete *C. cadaveris* genome sequence may therefore advance pangenome analysis of this species, especially in the context of tissue necrosis associated with human disease.

### Data availability.

The BioProject accession number for this genome, as well as that for many other human-associated bacterial isolates, is PRJNA549513. The RefSeq assembly accession number is GCF_020911725.1. The genome sequence was deposited in GenBank under the accession number CP076620. The base modification files are available with the GenBank accession and methylome analysis at REBASE under organism 49902 (http://rebase.neb.com/cgi-bin/onumget?49902). and methylome analysis is available at REBASE under organism number 49902.
